# Supplementary material for: A Global Screen for Assembly State Changes of the Mitotic Proteome by SEC-SWATH-MS
Source: Cell Syst. 2020 Feb 26;10(2):133–155.e6. doi: 10.1016/j.cels.2020.01.001 (PMC7042714; doi:10.1016/j.cels.2020.01.001)

Q6KCM7 | SCMC2_HUMAN | SLC25A25 APC3 KIAA1896 MCSC3 SCAMC2 UNQ549/PRO1106
Monomer MW [kDa]: 52.663 Monomer expected elution fraction: 46

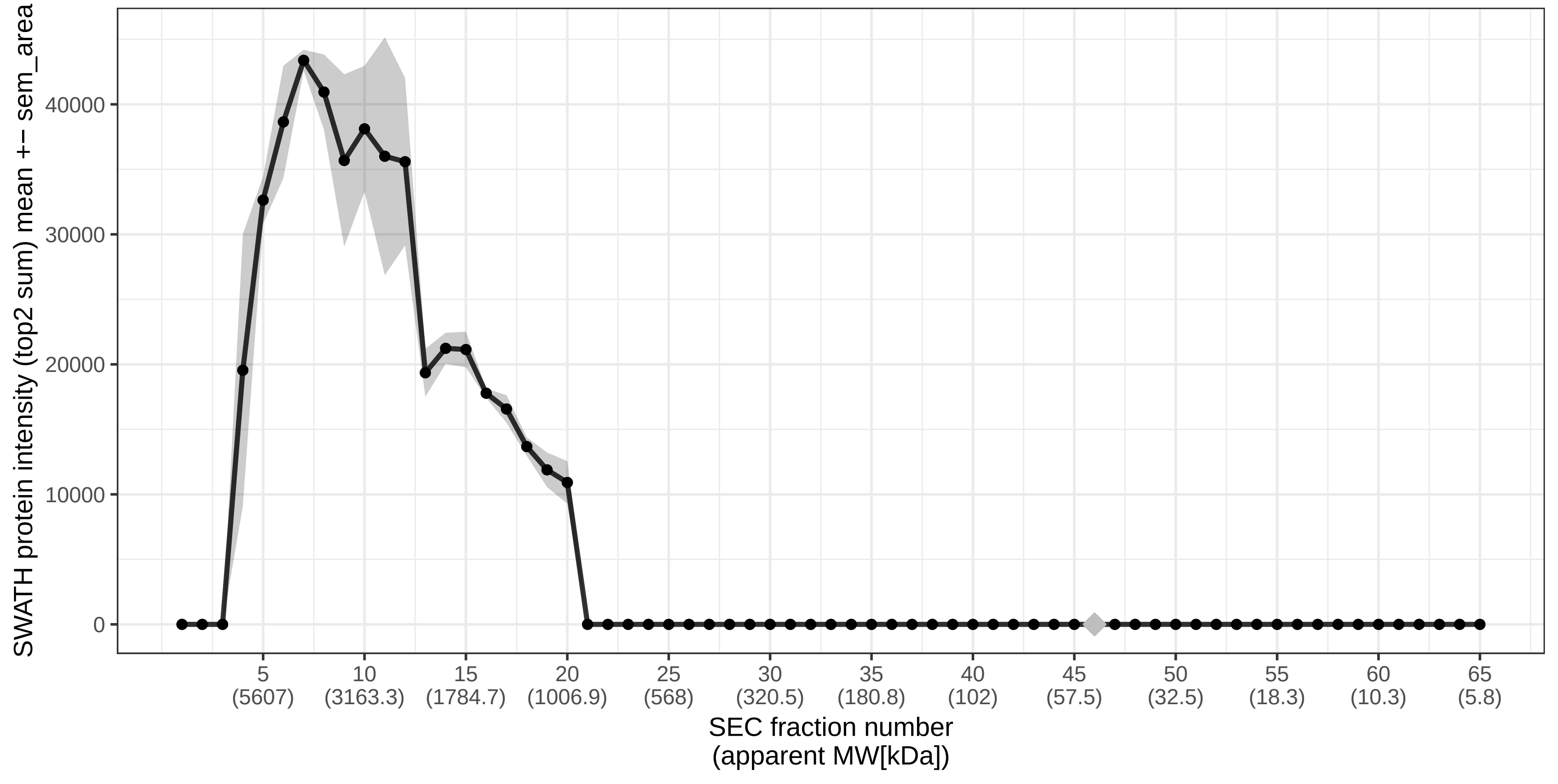

Supplement: Data S1. SEC-SWATH-MS Protein Chromatograms, Related to Figure 1 [file mmc6.zip › SECchrom_Q6KCM7_SCMC2_HUMAN_SLC25A25_APC3_KIAA1896_MCSC3_SC.pdf]
